# Eleven Reasons for Adaptation of Swedish Parenting Programs

**DOI:** 10.3389/frhs.2022.923504

**Published:** 2022-07-11

**Authors:** Kristoffer Pettersson, Pernilla Liedgren, Fabrizia Giannotta, Ulrica von Thiele Schwarz

**Affiliations:** ^1^School of Health, Care and Social Welfare, Mälardalen University, Västerås, Sweden; ^2^Department of Public Health Sciences, Stockholm University, Stockholm, Sweden; ^3^Procome Research Group, Medical Management Centre, Department of Learning, Informatics, Management and Ethics, Stockholm, Sweden

**Keywords:** parenting program, adaptation, fidelity-adaptation, implementation, sustainment, cultural adaptation, parental support, evidence-based intervention

## Abstract

While questions about adaptation and fidelity are of great concern in many implementation projects, less attention has been paid to reasons for adaptations that remain when evidence-based interventions (EBIs) are used in clinical and community settings. This study aims to explore reasons for adaptations that can arise when using parenting programs in a community setting. Seventeen individual interviews with providers were conducted and analyzed thematically, resulting in 11 reasons for adaptations organized into four separate areas: characteristics of group leaders (*supplementary skills and knowledge, preferred ways of working*), characteristics of families (*problem complexity, diverse or limited educational experience, non-parenting needs for support, colliding value systems*), group incidents (*criticism and challenges, excessive questions or discussions*), and didactic challenges (*lack of focus or engagement, limitations of the material, language differences*). The study shows that factors triggering adaptation and fidelity decisions continuously reappear in the provision of parenting programs in community settings. Knowledge about reasons for adaptation can be used to inform decision-making during implementation planning, as well as the sustainment of implemented interventions.

## Introduction

Several studies have shown that high fidelity (i.e., using interventions as initially designed) when implementing evidence-based interventions (EBIs) is related to improved outcomes. However, research also suggests that adaptations (i.e., thoughtful and deliberate modifications to the content or delivery of interventions) can lead to beneficial outcomes (1–3). These conflicting findings, and the accompanying debate surrounding fidelity and adaptation suggest that adaptation decisions are not yet fully understood.

Adaptations have been defined as the thoughtful and deliberate alteration of interventions with the goal of improving their fit with the target context (4, 5). For example, adaptations based on the client's cultural background can potentially increase the acceptability of the intervention (6, 7). Deliberate adaptations can also be made in response to contextual features that arise during implementation (4). However, adaptations can also be unplanned and made in a way that threatens treatment integrity, a process sometimes labeled *drift* (8). Because of this, it is recommended that adaptations are planned to ensure that the core components that make the EBI effective are preserved (9). Thus, adaptations can be planned but still be inconsistent with core components of the intervention, and adaptations can be unplanned and made in ways that are consistent with core components. Without a clear and nuanced understanding of challenges and opportunities in the local context, successful adaptations are difficult to achieve, thereby increasing the risk of unintended drift from the core components of the EBI. Even with a solid understanding of the initial challenges, there might still be reasons for adaptation that are not anticipated. From this, it follows that successful adaptation requires a fine-grained understanding of all the factors that have the potential to influence modifications.

Although research on reasons for adaptations is limited, factors at the client, provider, organization, and broader sociopolitical levels have been reported. This includes client population characteristics, such as participant dissatisfaction (10), cultural background (6), and perceived needs (11). Providers' attitudes toward EBIs (12–14) and organizational factors such as limited resources (10) and lack of time (15) have also been reported. More broadly, sociopolitical factors, such as financial allocations or other political actions, might influence the likelihood of adaptation through indirect means (5). In recent years, implementation researchers have developed frameworks to support consistent reporting of adaptations and modifications (1, 5, 16, 17), and when needed, use them to make better adaptation decisions (9). The framework with the most comprehensive guidelines for reporting reasons for adaptations to EBIs is the expanded Framework for Reporting Adaptations and Modifications-Enhanced (FRAME) developed by Stirman et al. (5). Taken together, previous research on reasons for adaptations and frameworks like FRAME provides a comprehensive list of possible reasons to consider. However, it is not clear if these reasons are especially relevant to consider during the planning of implementation efforts, or if they remain to be managed during routine practice.

This study aims to explore reasons for adaptations that can arise when using parenting programs in a community setting. Studies focusing on reasons for adaptation of parenting programs are rare; the only examples we found in the literature focus on adaptations made for cultural reasons (3, 18–20). These studies focused on reasons for adaptation that were planned before implementation. By exploring reasons for adaptation that are present in, albeit not necessarily exclusive to, the sustainment phase, we intend to increase the understanding of reasons for adaptation that might be difficult to anticipate earlier in the implementation phases, thereby adding knowledge about factors that could influence unplanned adaptations when using parenting programs in practice.

## Methods

The study is an exploratory interview study focusing on group leaders of parenting programs. These programs make a suitable case for studying reasons for adaptation, since they (1) are well disseminated in practice settings and thereby readily available for study; (2) are delivered with an expectancy of fidelity, which naturally raises the question of adaptation; (3) have a well-established evidence base; and (4) are provided by several categories of professionals, which increases the scope and generalizability of the study (21).

### Studied EBIs

Parenting programs are preventive psychosocial interventions targeting several childhood phases, from childhood to the upper teenage years. These programs are provided nationally in most of Sweden's municipalities. Programs are usually delivered in a group format, led by professionals with special training in providing these interventions. The evidence-based parental programs that are among the most widely disseminated in Sweden are All Children in Focus (22), Comet (23), Triple P (24), COPE (25) and Connect (26). Although there are differences between programs, they all focus on teaching parents fundamental parenting skills to reduce coercive parenting, strengthen parent–child relationships, and reduce externalizing problems (27).

### Recruitment and Participants

Municipalities in Sweden are the primary providers of parenting programs with a national reach. Thirty out of Sweden's 290 municipalities were selected. We used stratified purposeful sampling (28) to ensure that municipalities of all sizes, both rural and urban, were included in the sample. Once information on size and geographical location was identified from public records, municipals' websites were used to gather contact information and the type of parental programs offered. Ten of the 30 contacted providers did not respond. An initial meeting was held with the managers of the 20 provider organizations that responded. Eight of these agreed to participate in the study and received information that they distributed to professionals working as group leaders for parenting programs in their organizations. Eighteen professionals agreed to participate; of these, 12 were invited to interviews, ensuring representativeness from all parental programs included in the study. Later, five additional group leaders with experience working with non-native Swedish parents were included to provide further examples of reasons for adaptations tied to cultural factors.

A total of 17 group leaders from various professions participated in the study (Table 1). Average age was 52.3 (SD = 9.00, range = 37–65), average experience as group leaders for parenting programs was 8.6 years (SD = 5.18, range 3–20) and average number of groups conducted was 15.6 (SD = 9.97, range 3–35). The parenting programs represented were Triple P (*n* = 6), All Children in Focus (*n* = 6), Connect (*n* = 2), COPE (*n* = 2), and Comet (*n* = 2). Eight group leaders had training in several parenting programs, but all expressed a clear preference for the program listed in Table 1. All participants had previous experience working with children and families in their primary professional roles. After the second round of recruitment, eight participants reported having experience working with non-native Swedish parents. Two of the participants were also supervisors and teachers of parental programs.

**Table 1:** Background characteristics of the group leaders included in the study.

**No**.	**Profession**	**Group experience**	**Years of experience**	**Program**
1.	Health adviser	5–10	5–10	All Children in Focus
2.	Preschool teacher	>30	5–10	Triple P
3.	Preschool teacher	20–30	5–10	Triple P
4.	Preschool teacher	<5	5–10	Triple P
5.	Preschool teacher	20–30	10–20	Triple P
6.	Preschool teacher	<5	>20	All Children in Focus
7.	Preschool teacher	<5	5–10	Triple P
8.	Preschool teacher	5–10	<5	Triple P
9.	Bachelor social work	>30	10–20	All Children in Focus
10.	Bachelor social work	10–20	10–20	All Children in Focus
11.	Nurse	10–20	5–10	Connect
12.	Health adviser	20–30	10–20	Connect
13.	Bachelor social work	10–20	<5	All Children in Focus
14.	Bachelor psychology	20–30	5–10	Comet
15.	Bachelor sports and community	10–20	<5	COPE
16.	Pre-school teacher	20–30	10–20	Comet
17.	Bachelor social work	10–20	5–10	COPE
		15.6 (SD = 9.97)	8.6 (SD = 5.18)	

### Data Collection

Semi-structured individual interviews were conducted with all participants. Interviews were performed by one of the authors (KP) through an online meeting platform (Zoom) and recorded locally using third-party software (VideoSolo). The average length of the interviews was 36 min (ranging from 24 to 44 min).

The authors developed the interview guide collaboratively, and the questions were formulated based on previous experience conducting similar qualitative studies on fidelity and adaptation (29). The interview questions focused on identifying circumstances that might lead practitioners to adapt to parental programs. It was assumed that some reasons for adaptation would be program-specific. Still, since we aimed to study reasons arising across all programs, we primarily directed questions to shared program characteristics. Questions were asked about potential obstacles to fidelity, what makes these situations hard to handle, and what might make them prevalent. Example of questions that was used: “In what kinds of situations do you hesitate about what to do to adhere to the program?” and “Are there any specific circumstances that might make adaptations more likely?” The questions were also aimed at identifying reasons for adaptations that were more common among non-native Swedish parents. Some questions used for this purpose were “How is working with non-native Swedish parents different?” and “What kinds of situations might make it easier/harder to adhere to the program when working with non-native Swedish parents?”

The study was approved by the Swedish Ethical Review Authority (Dnr 2021-00832). Participants were given an oral and written description of the purpose of the study, what participation entailed, that no data that would identify them as individuals would be reported, and that they could withdraw their consent at any time without further explanation. All participants gave written informed consent before the interviews began.

### Analysis

All interviews were transcribed verbatim and then analyzed using thematic analysis (30, 31). A theme was defined as any circumstance that could lead to the adaptation of programs. These circumstances were assumed to be specific to the provider context, events in the groups, general attributes of working with parenting programs, and other external or internal processes influencing the group leaders.

All transcribed interviews were read, and a first coding was done in which all data units (verbal expressions by group leaders) relevant to the study were extracted from the material. Next, data units that seemed to be connected to the same phenomenon were grouped. These groups were assumed to represent initial themes that were later developed and refined as the analysis progressed. Next, the data units in each main theme (areas) were divided into subthemes. Each theme was then given a provisional label and description, after which each interview was reread to confirm the analysis. Each theme was provided with a final label and description in the last step, with accompanying quotes from the material.

Since the goal of the analysis was to openly explore the reasons for adaptation of parenting programs, the analysis was inductive; no models or theories were used to inform the grouping of data units into themes. The coding was done by KP, analyses were made in collaboration between KP and PL, FB and UvTS acted as the auditors of the results and their interpretation.

## Results

**Figure 1:**
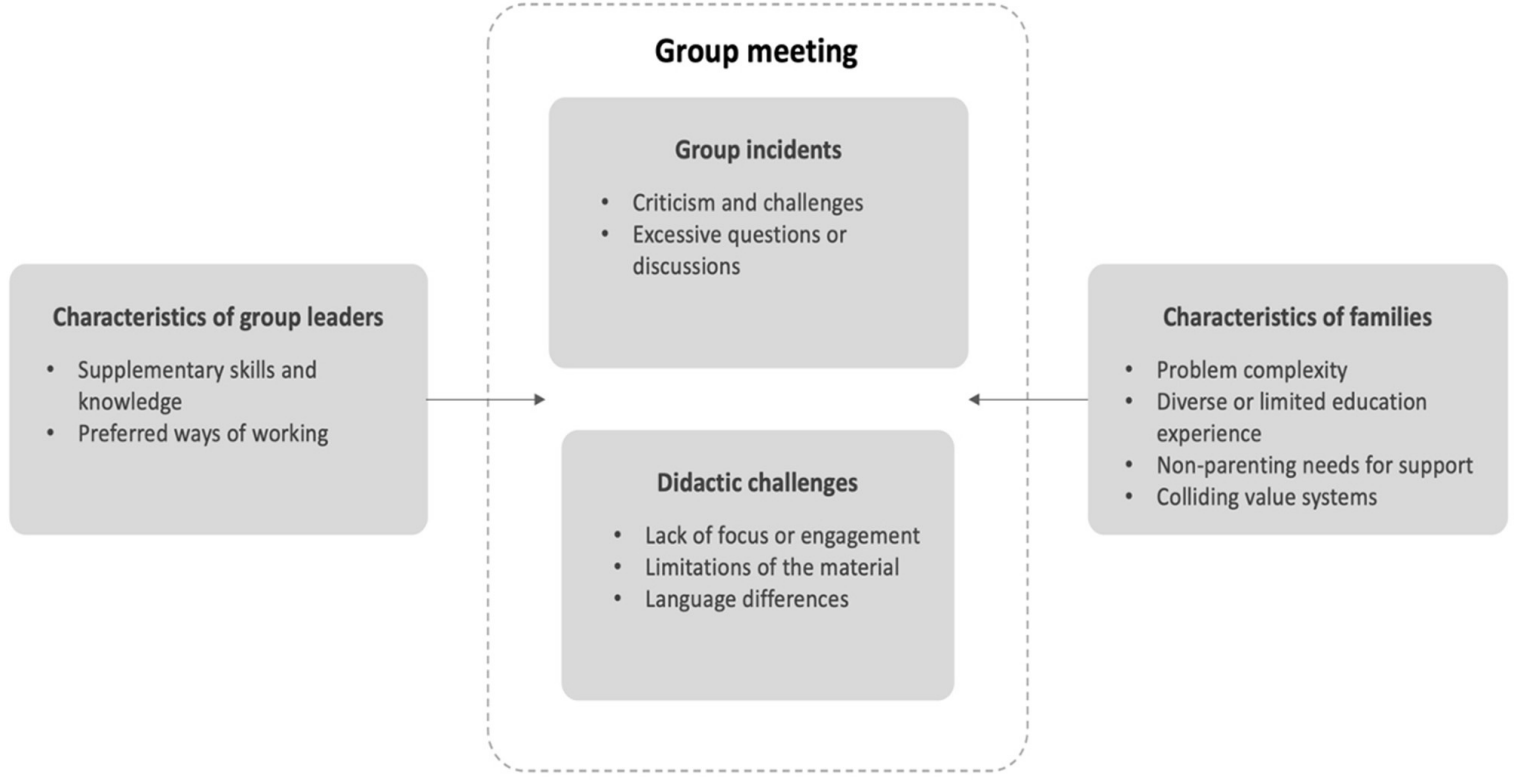
Identified reasons for adapting parental programs. *Characteristics of group leaders* and *families* summarize reasons for adaptation brought to the group meeting by each party. In contrast, group *incidents* and *didactic challenges* outlined reasons arising during the group meeting.

Eleven reasons for adaptations were identified and organized into four areas: *characteristics of group leaders, characteristics of families, group incidents*, and *didactic challenges* (Figure 1). Group leader characteristics and family characteristics summarize the reasons for adaptation that each party brings to the interaction, depicted as arrows going to the middle of Figure 1, illustrating that these reasons for adaptation manifested in session. Didactic challenges and group events summarize reasons for adaptation that were said to arise during the delivery of the program.

### Characteristics of Group Leaders

Several group leaders describe how *supplementary skills and knowledge* from other areas of their life, including work roles and different life experiences, might affect their approaches to leading parenting groups. They also describe how their *preferred ways of working* can influence the content and style of program delivery.

#### Supplementary Skills and Knowledge

The group leaders have had several other professional and private commitments apart from parenting programs. Skills and knowledge from different contexts sometimes provoke difficulties adhering to the program, either by affecting their stance as group leaders or how they deal with specific topics. For example, one participant describes a habit of focusing on individuals instead of groups in her usual profession:

*If someone says something interesting and you go too much into detail, that*'*s a kind of risk when you are used to working with individuals. (Group leader No. 6)*

Because of this, this group leader sometimes struggles to adhere to parts of the manual that promote group interaction. In some cases, group leaders also have specific knowledge that goes beyond the program but is still relevant for parents:

*Then I give them concrete tips, since I also work at a preschool and know which way they should go. (Group leader No. 2)*

Professional experience can also be a reason for group leaders to focus on specific topics not covered in the program. This includes previous work roles and experience working with other parental programs, as well as personal experiences. Some examples that are mentioned by the group leaders include: focusing on health-related subjects as a result of working in public health, using concepts from other parenting programs, and drawing on personal experiences of taking care of children.

#### Preferred Ways of Working

Some group leaders explain that their preferences can conflict with the content of the program and thereby influence them to make adaptations. These reasons are connected to particular subjects not included in programs, for example, interventions to strengthen the relationship among parents:

*You know, this is personal development. I let them draw a line and then they get to write about their partner as well, what they are proud of. It becomes a bit like relationship-building as well. (Group leader No. 1)*

Several group leaders describe how preferred ways of interacting with others can affect their way of leading parenting groups. Most preferred giving more explicit guidelines and interacting more personally than prescribed in the manual:

*I talk a lot, as you can tell. So I try to hold back, and I know that I should listen more and lead the conversations they have amongst themselves. (Group leader No. 1)*

Reasons for adaptations tied to preferences can be understood as group leaders' struggles with adapting to the content and style of the parenting program they apply, which sometimes result in adaptations.

### Characteristics of Families

Group leaders describe several reasons for adaptation that stem from family characteristics, including their assessment of families' *problem complexity*, parents having *diverse or limited education experience, non-parenting related needs for support*, and *colliding family values*.

#### Problem Complexity

All group leaders describe a continuous effort to assess families' problems and needs indirectly, and this assessment is one of the main reasons for adapting the program. These assessments are sometimes broad, taking several kinds of factors into account:

*You have to adapt the content according to the children*'*s age, their challenges, intellectual capabilities actually, depending on who the parents are. (Group leader No. 4)*

This statement echoes the assumption that several other group leaders seem to hold that families have different problems and needs simply because everyone is unique. Others focus more strictly on problem severity, which might be a reason to either terminate or extend support:

*So if a need for support emerges that there isn*'*t room for, we let them know there are other ways of handling it, that we can help with that, make sure it gets addressed. Sometimes I*'*ve arranged individual sessions. (Group leader No. 13)*

The group leaders mentioned several other problem kinds and degrees of severity: psychiatric symptoms (in children and adults), general exhaustion among parents, and children with a history of disruptive behavior.

All group leaders seem to agree that problem complexity is a justified reason for adapting programs, although the interpretations of problem types and their severity differ between them.

#### Diverse or Limited Educational Experience

Parents' education levels naturally range from almost no school experience to highly educated, influencing group leaders to carefully consider their style of delivery. One of them describes it this way:

*Some are illiterate, and some have college degrees, so yes, it can take some time to explain every step. If only I had a homogeneous group, there*'*d be adherence (Group leader No. 5)*

Although diverse education levels can influence adaptations, limited education levels among parents can also be a problem. The effects of education levels extend further than simply a lack of conceptual knowledge would suggest. Several group leaders mention that parenting programs implicitly assume specific skills typically acquired through school experience, such as attentive listening, group discussions, following a plan, reflecting, drawing conclusions, and problem-solving. One group leader describes the situation like this:

*It*'*s a matter of being in a group, cooperating, discussing things with others, and then going back to reflect. I mean, it*'*s a kind of method that is a bit like school. (Group leader No. 13)*

When several of these skills are missing, group leaders might need to adjust the content and intervention procedures to keep everyone involved. These reasons are typically described by professionals working with immigrant families, but others also mention that groups with native parents can provoke the same concerns.

#### Non-parenting Needs for Support

Working with immigrant families, it is not unusual for parents to use the group setting to handle welfare support applications, discuss school-related issues, or other government communications:

*Sometimes when we start, a parent needs help filling out some papers. (Group leader No. 11)*

For group leaders, this is cause for some ambivalence, since they want to be of service and help parents deal with everyday problems that could affect their ability to function as parents. One of the group leaders describe it this way:

*It*'*s also a bit like a civics lesson for them, and we become a sounding board for so many more issues than just parenting. So, in a way, it*'*s almost like the parenting strategies take second place, compared to when we work with Swedish-speaking parents. (Group leader No. 15)*

However, these considerations take time and focus away from the parenting program. In some cases, group leaders handle this concern by assigning extra time to these extraneous topics, while others prevent these discussions by clearly pointing out the purpose of the meeting.

#### Colliding Value Systems

Parenting can be loaded with values that are easy to assume to be general, even though they are tied to cultural background, ethnicity, and group belongings. Working with immigrant families highlights how some of these assumptions need to be considered. Group leaders describe a range of topics that can become problematic, such as teenagers arguing about meeting a partner or lack of respect for authorities, examples that seem unrealistic from the Middle East or Somalian perspective:

*There are families from the Middle East or Somalia. Some situations aren*'*t really present there, so that*'*s when we try to take away and add things (Group leader No. 11)*

Another topic that raises questions of values is views about authority and punishment. Several group leaders describe that a common belief among immigrant families, especially those from the Middle East, is that discipline is necessary for children to learn. Although one of the group leaders points out that views on punishment can be cumbersome to handle, even in groups with native Swedish parents, it is a more prominent issue in immigrant families:

*The usual thing is that there should be some kind of punishment. If the child has done something wrong, they should receive some kind of consequence, otherwise, they won*'*t learn. This is actually not only a thing among immigrants, it*'*s quite common. But consequences are almost identical to punishment in immigrant families. (Group leader No. 5)*

Conflicting views about parental authority are also a common source of insecurity among immigrant families. According to the group leaders, parents tend to be unsure about Swedish law and regulations in this area:

*It*'*s really hard to come to a new country and not know how things work. The first thing they hear is that it*'*s illegal to spank children, otherwise the social services will come and take your kids. They don*'*t know, they lose their footing. They don*'*t know how to be parents. (Group leader No. 5)*

None of these issues are discussed in parenting programs, but since they arise in working with immigrants, group leaders continuously need to handle them. Thus, colliding family values becomes a common reason for adaptation, especially when working with immigrant families.

### Group Incidents

Some of the challenges described by group leaders have to do with the group situation and what arises during meetings. Generally, group leaders try to ensure that the group setting promotes learning and adherence to the program. However, *excessive questions or discussions* can provoke interactions that take time and thereby challenge fidelity. Group leaders also describe how direct *criticism and challenges* can be difficult to handle without adapting the program.

#### Excessive Questions or Discussions

All group leaders bring up their ambivalence about the number of questions and discussions during group meetings. On the one hand, questions are seen as signs of parents' engagement, which they view as beneficial for learning. However, on the other hand, questions can also lead astray, making it harder to focus on the material. One of the group leaders express this ambivalence as follows:

*Absolutely right, they should talk. And then, as group leader, you*'*re always in two minds; is it good that they talk? (Group leader No. 10)*

Sometimes, group leaders sense that discussions are productive and let them continue for a while, even if this means that they will struggle to complete the content for the day:

*If they have a good and productive discussion, we might skip certain parts. (Group leader No. 3)*

In other cases, discussions are not productive, but group leaders let them continue to promote collaboration and a better group climate.

#### Criticisms and Challenges

Several group leaders describe difficulties handling parents' opinions about them or the program. In some instances, their authority is directly challenged, making it difficult to adhere to the program content. In other cases, parents are skeptical about some part of the program content. This problem is partly due to group dynamics, which can make group meetings an arena for power struggles. For example, one of the group leaders describe the group situation using expressions such as *strong forces* and *challenge me*:

*There might be powerful, I mean, strong forces in a group, more influential parents who are looking for ways to challenge me a bit more. (Group leader No. 6)*

Depending on the group leader, these forces can provoke adaptation responses. However, they can also be opportunities for reflection and learning if group leaders handle them effectively.

Other group leaders mention that criticism can sometimes connect to their struggles with certain concepts. Their confusion or insecurities around specific topics might shine through, open them up for criticism that provokes anxiety and make it harder to stay the course. One of the group leaders give an example of being questioned when discussing the concept of self-esteem:

*When we discussed self-esteem, someone started to question the concept, what it meant. Then I felt that we started to move away from the topic we were discussing. (Group leader No. 8)*

Nonetheless, most group leaders view these challenges as natural and part of leading parental groups. They also mention that collaborating with another group leader can help, making it easier to handle criticism and challenges without straying from the program.

### Didactic Challenges

Group leaders describe different levels of concern regarding the quality of the presentation and delivery of the programs. Some of these concerns are directly related to the perceived *limitations of the material* accompanying the program. Other challenges arise from the interaction of content and attending parents' reactions, such as their *lack of focus and engagement* and trouble understanding due to *language difficulties*.

#### Limitations of the Material

Several group leaders bring up their perceptions about the limitations of the teaching material or the methods described. These problems are largely specific to the interventions, even though some overlap occurs. When group leaders notice the limitations of the material, they are more likely to make adjustments. For example, they might remove parts that they perceive to be less critical or somehow not presented well:

*Sometimes I actually skip the research comments. I find the research to be particularly hard to fit in at the right moment. (Group leader No. 1)*

Another limitation that group leaders report relates to how the material is presented. The material might be unnecessary, repetitive, or cumbersome. Group leaders also mention specific problems, with parts of the material being outdated. One group leader brings up this point when describing the limitations of Comet:

*My feeling is that Comet needs to be updated. Things are changing and new knowledge is being developed. So, I think the adaptations that are being made, at least the ones I can think of, probably have to do with the program starting to feel a bit old. (Group leader No. 17)*

Even though several group leaders are critical of aspects of the accompanying material, they also emphasize that overall, the material is helpful and supports them in adhering to the program.

#### Lack of Focus or Engagement

All group leaders describe a general ambition to involve parents in a collaborative and engaging teaching environment. This is viewed as a prerequisite for effective learning and participation in groups. As the program is being delivered, parents' degree of focus or engagement during group meetings is one of their primary ways to assess if they grasp the content. Many group leaders say they are willing to adapt the program to increase parental involvement, as in the following example:

*When I feel there*'*s a need to explain things further. When you feel that the group isn*'*t on the same page as you. When we*'*re standing around talking about something in the manual and you feel like “What is it they want?” That*'*s when you might need to go outside the manual a bit. (Group leader No. 12)*

Group leaders also reflect on how they respond to changes in engagement. When a parent has been quiet for a long time and suddenly seems more alert, group leaders might use that opportunity to make a stronger connection and get that parent more involved in the content. In these cases, group leaders are not focused on adherence.

Some group leaders are also sensitive to aspects of the situation that might make it harder to focus on program content. One of the group leaders brings up lack of sleep as an example of this problem:

*When they haven*'*t slept in weeks and are tired to death, then it*'*s not possible to go through everything, you must minimize. (Group leader No. 4)*

Other group leaders also mention that certain parts of the program might provoke strong emotions. In these cases, they are ready to adapt the session's content to not overwhelm parents to the point of failure to process the information.

#### Language Differences

Among the group leaders who work with immigrant parents, language differences can be an important reason for adaptation. Difficulties range from general aspects, such as working with an interpreter, to specifics, such as finding the correct way to convey certain program concepts and ideas. When using an interpreter, the group leaders agreed that role-playing is cumbersome and sometimes even impossible:

*It*'*s not possible to role-play with an interpreter. So, we had to get rid of all the role-playing activities. (Group leader No. 15)*

Some group leaders working with immigrants deliver the program in the recipient's native language. For them, specific concepts can be of particular interest and provoke reflections about the nuances of words and their translations. For example, one Arabic-speaking group leader describes her difficulties explaining the differences between *punishment* and *consequences*, a nuance not often expressed in lay Arabic:

*There*'*s a difference between consequence and punishment. And in the Arabic language, one usually says ”punishment.” So how to explain it so that it becomes “consequence” instead? That*'*s a challenge. They are so similar in Arabic. (Group leader No. 5)*

Another Arabic-speaking group leader struggles to explain the concept of empathy, and through her effort, she resorts to quoting the Quran to convey its meaning.

Generally, working with immigrant parents who are not fluent in Swedish tends to slow the pace of group meetings, making it hard to complete the whole program within the specified time available.

## Discussion

This study aimed to explore reasons for adaptations that can arise when using parenting programs in a community setting. We identified four areas in which reasons for adaptation were present and 11 specific reasons for making adaptations to parental programs. The findings add knowledge about the factors contributing to adaptation during the sustainment phase of parenting programs. Any identified reasons stemming from group leaders, parents, group interaction, or didactic challenges could potentially influence group leaders to modify the intervention. Thus, reasons for adaptation seem to be present, even in already implemented programs where potential barriers have supposedly already been worked through.

Group leader and family characteristics were conceptualized as reasons being brought into the group meeting by each respective party. Similarly to previous research which has found that adaptations can be made to meet perceived needs (11), or in response to cultural factors (6), group leaders in the present study described factors related to the participants' situation, specifically *problem complexity* and *colliding value systems*, as reasons for making adaptations. However, the group leaders also spoke about the influence of their own experiences and preferences as possible reasons for adaptations. These reasons seem to extend beyond attitudes toward EBIs (12), giving way to adaptations, not because of any aversion toward EBIs, but because group leaders view their experiences, skills, knowledge, and perspectives as valuable additions to the programs. Although some research suggests that clinicians' personality traits can influence their professional orientation and preferred ways of working (32), and that clinicians' training and openness toward EBIs affect fidelity (33), the importance of these attributes for the topic of fidelity and adaptation dilemma has not been fully explored, meriting further consideration in future studies.

Previous research has shown that one of the main reasons for adaptation is a lack of time (15). This raises the question of whether time concerns are a reason in and of themselves or a result of having to manage circumstances that arise during delivery. Group meetings that produce the kinds of group events and didactic challenges identified in this study can potentially challenge group leaders' time management. Likewise, handling parents with complex problems or other needs not directly met by the program might distract in ways that make adherence to specified time constraints harder. These findings suggest that the lack of time might include several kinds of influences that can be perceived as related to time, but actually, be based on other factors. Some group leaders will not find these situations challenging, while others will struggle to stay the course. From an implementation perspective, these kinds of events could give rise to modifications that are unplanned/reactive (5), with the potential for drift to occur (8). However, the distinction between reasons that provoke adaptation and drift is not always easy to make. Whether a situation generates fidelity, adaptation, or drift depends on the practitioner's ability to correctly notice things of importance and their ability to handle the situation with a clear outcome in mind. Receiving criticism, for example, could be an opportunity for resolving misunderstandings or changing courses to avoid certain topics, depending on how the group leaders perceive the situation and their ability to handle it. In all cases, the findings suggest that “lack of time” as a reason for adaptation requires further scrutiny, as it is likely not a cause but a consequence of events that group leaders must manage.

The need for cultural adaptation of preventive interventions has been well recognized in the literature (6, 34), and several studies have pointed to the value of adapting parenting programs to minority groups (18, 19, 35–37). One challenge identified here is that one of the reasons for cultural adaptations is colliding value systems, which might interact with core components of the program, such as differing perspectives on punishment and the degree of parental authority. This suggests a challenge not likely resolved through program adaptations, yet that still is likely to take up time and effort from the group leader, potentially leading to other reasons for adaptations (e.g., challenges and efforts to add components to further justify and explain non-authoritarian parenting). This problem could potentially be solved by further research that identifies how cultural factors interact with the core components of programs, moving from reactive adaptations or drift toward proactive and planned adaptations.

What complicates the matter is that cultural adaptations have more dimensions than those related to differences between the majority and minority populations within a country. First, the group leaders reported that some parts of the programs felt outdated. This could reflect a cultural development over time, such that subtle changes in language, clothing, etc. reduce the appropriateness of the material. Second, many parental programs developed in other countries and used in Sweden undergo cultural adaptations at the program level. For example, Swedish culture emphasizes non-authoritarian parenting in both law and values (38). Since corporal punishment was made illegal in Sweden 1978, the practice has become increasingly rare (39), although it still exists, albeit at a lower frequency compared to other countries (40). In the United States, corporal punishment has been declining as well, but still, 37 % of children are subjected to some form of corporal punishment (41). In Kenya, 76.4% of parents, and in Iraq, 67.2%, agreed that the beating of children could be justified (38). In perspective, Swedish parents tend to instead use restrictions and verbal control to manage their child's behaviors (42). In line with Swedish parenting values, program components customarily in other Western cultures, such as time out, are not culturally accepted in Sweden. The adaptation of parenting programs to Swedish parenting values has not been studied scientifically, but it is likely that parenting programs given in Sweden are adapted in practice as a result of group leaders' own views on parenting. Minority groups from cultures with more authoritarian parenting styles are thus exposed to a program that, from their perspective, likely is at the other end of the authority spectrum. In effect, topics related to parenting styles might be brought to the forefront of parenting programs due to the sharp contrast between Swedish non-authoritative values and those held by families raised with more authoritarian parenting styles. Third, minority groups, such as immigrant families, are not exclusively characterized by their values. They are also in the midst of figuring out a new society, learning a new language, and finding ways to support their family. Like the previously discussed topic of time constraints as a reason for adaptations, cultural values should not be used as a catch-all explanation for every challenge to fidelity present in working with this population. That said, it is also clear that there are several reasons for adaptation that arise when working with immigrant parents. This could potentially have a compounding effect that makes it even harder for group leaders to adhere to the program.

As noted in the introduction, the most comprehensive framework for reporting on reasons for adaptation to EBIs is the expanded Framework for Reporting Adaptations and Modifications-Enhanced (FRAME) developed by Stirman et al. (5). Although the present study did not use FRAME to guide the exploration, we note that there are similarities in our findings and the reasons for adaptation mentioned in this framework. FRAME lists a total of 42 reasons for adaptation that is divided into four areas: sociopolitical, organization/setting, provider, and recipient. The reasons for adaptation that we identified as stemming from *characteristics of group leaders* are similar to the *provider* area in FRAME. The reasons identified as stemming from *characteristics of families* are found in the area of *provider*, with the addition of some *sociopolitical* factors related to family values and norms. The area in FRAME labeled *organization/setting* has some similarities to *group incidents* in our conceptualization, although our focus was confined to the setting where the interventions were delivered. The only findings from our study that do not seem to have a direct relation to FRAME are the reasons grouped in *didactic challenges*. Although reasons related to language are mentioned in FRAME under *provider* and *recipient*, there are no mentions of *limitations of the material* or *lack of focus or engagement*. This is probably due to these factors being quite specific and tied to the specific kinds of EBIs in focus, although further research is needed to confirm this hypothesis.

### Methodological Considerations

This qualitative study used thematic analysis (30, 31) to explore reasons for the adaptation of parenting programs, thereby establishing the credibility of findings due to the use of an established procedure for thematic analysis. Through the study's design, care was taken to ensure trustworthiness with respect to the criteria of sound qualitative research (43). To further increase credibility and dependability, we utilized an iterative process of discussion between the authors during the coding and development of themes.

Using a qualitative approach, we identified reasons that would have been hard to explore by other means. It should be noted that the group leaders found the topic difficult to discuss. One possible explanation for these difficulties is that adaptation is taboo in some contexts. Throughout the interviews, group leaders repeatedly hesitated and sometimes even used phrases indicating that they were making confessions about mistakes. Another possible explanation is that discussions about adaptation are uncommon, especially in service settings that emphasize fidelity. It might be that group leaders simply had not approached the topic in a focused manner before. Given these difficulties, it is possible that extended or repeated interviews would yield even more nuanced findings. Nonetheless, we managed to identify a set of possible reasons for adaptations to parenting programs that could be further explored in future studies.

Regarding transferability, we included established and well-known parenting programs that have been the focus of carefully planned implementation efforts in Swedish social welfare systems. We aimed to include group leaders from various professions working in both large and small municipalities in Sweden. Our focus during the interviews was to explore general reasons for adaptation rather than those specific to the programs used. Thus, the results speak to general phenomena in working with parenting programs. However, *limitations of the material* should not be taken as transferable to other programs, since these comments were quite specific compared to the rest of the results. Also, as noted above, the parenting style in Sweden is usually non-authoritarian (38, 44). Thus, some reasons for adaptations may stand out more in this sample than others but are likely to be more of a matter of magnitude than type.

### Implications

The results from our study show that, even in well-implemented parenting programs, there are still features that could be improved to better fit the target population and local contexts. Some of the reasons we identified could be used to proactively plan adaptations, as recommended in the implementation literature (9, 45). For example, providers of parenting programs could implement assessment routines to minimize problems related to mismatches in problem complexity or educational experiences among parents. In working with immigrant families, non-parenting issues can be handled separately to increase the focus on parenting during the delivery of parenting programs. The training of group leaders could also incorporate explicit discussions of how supplementary skills and knowledge, or preferred ways of working might affect program delivery, including clear guidelines of what would constitute acceptable divergences from the program instead of a sole emphasis on fidelity. Without taking these issues into account, group leaders are left to deal with these challenges as they arise, with an increased risk of unplanned/reactive adaptations or drift.

Finally, this study points to the need for the continuing development of parenting programs, even in cases where extensive efforts have been made to disseminate interventions. This is in line with the dynamic sustainability framework, which outlines that not even well-supported EBIs should be considered final once they are disseminated and spread (46). Continuous improvement in programs may include updating teaching materials to keep up with developments in society and general knowledge development. Still, it may also address more fundamental issues, such as changes in the target population's needs, such as those of immigrant families in Sweden, which may include addressing the collision of implicit values systems and the need for guidance in a new society. Thus, program development may consist of changes related to the intervention to better meet current and emerging needs. In this regard, EBIs must meet a complex web of values related to multiple stakeholders (47). With evolving EBIs, there is also a need for systematic ways to continuously track the impact of EBIs in practice in line with measurement-based care (48). As such, this reflects a shift in the research process, moving from a one-way road from the development and evaluation of EBIs to a two-way street of practice-based research as well as research-based practice (49).

## Conclusion

Even in well-implemented programs, there are still reasons to adapt evidence-based parenting programs. This puts providers in decision-making situations that could either result in contextual adaptations to retain or regain fidelity or adaptations to the programs (47). However, this situation is often unclear, even to group leaders. Group leaders must be aware of these decision processes before the kinds of structured, rational decisions that the literature advocates for can be made. There are also challenges to fidelity that will remain throughout the delivery of programs, which suggest that rather than only managing adaptations, group leaders need to be better prepared to autonomously assess whether features of the program need to be adapted to better fit the target population and local contexts, thereby making the decision-making process more explicit and conscious. This issue must be further researched to better understand the circumstances in which unwanted modifications could occur and those in which adaptations are justified.

## Data Availability Statement

The datasets presented in this article are not readily available because the study used qualitative data generated from interviews. Anonymous samples of the datasets used and analyzed during the study are available from the corresponding author on reasonable request. Requests to access the datasets should be directed to kristofferpettersson83@gmail.com.

## Ethics Statement

The studies involving human participants were reviewed and approved by the Swedish Ethical Review Authority (Dnr 2021-00832). The patients/participants provided their written informed consent to participate in this study.

## Author Contributions

KP collected data from interviews with participants, and PL, FG, and UT acted as the auditors of the results and interpretation. All authors were involved in the conceptualization of the research problem, research design, data interpretation, and contributed to the final writing process. All authors contributed to the article and approved the submitted version.

## Funding

This study received research funding from the Swedish Research Council for Health, Working Life and Welfare (Forte) (Reference No. 2020-01223). The funder has no role in the design and conduct of the study, including the collection, analysis, and interpretation of the data and the reporting of findings. The content is solely the responsibility of the authors and does not necessarily represent Forte's official views. Open access funding was provided by Mälardalen University.

## Conflict of Interest

The authors declare that the research was conducted in the absence of any commercial or financial relationships that could be construed as a potential conflict of interest.

## Publisher's Note

All claims expressed in this article are solely those of the authors and do not necessarily represent those of their affiliated organizations, or those of the publisher, the editors and the reviewers. Any product that may be evaluated in this article, or claim that may be made by its manufacturer, is not guaranteed or endorsed by the publisher.

## References

[1] Cooper BR Shrestha G Hyman L Hill L. Adaptations in a community-based family intervention: replication of two coding schemes. J Prim Prev. (2016) 37:33–52. 10.1007/s10935-015-0413-4 26661413

[2] Sundell K Beelmann A Hasson H von Thiele Schwarz U. Novel programs, international adoptions, or contextual adaptations? Meta-analytical results from German and Swedish intervention research. J Clin Child Adolesc Psychol. (2016) 45:784–96. 10.1080/15374416.2015.1020540 25864716

[3] van Mourik K Crone MR de Wolff MS Reis R. Parent training programs for ethnic minorities: a meta-analysis of adaptations and effect. Prev Sci. (2017) 18:95–105. 10.1007/s11121-016-0733-5 27882498 PMC5236066

[4] Moore G Campbell M Copeland L Craig P Movsisyan A Hoddinott P. Adapting interventions to new contexts-the ADAPT guidance. BMJ. (2021) 374:n1679. 10.1136/bmj.n1679 34344699 PMC8329746

[5] Wiltsey Stirman S Baumann AA Miller CJ. The FRAME: an expanded framework for reporting adaptations and modifications to evidence-based interventions. Implement Sci. (2019) 14:1–10. 10.1186/s13012-019-0898-y 31171014 PMC6554895

[6] Castro FG Barrera M Martinez CR. The cultural adaptation of prevention interventions: resolving tensions between fidelity and fit. Prev Sci. (2004) 5:41–5. 10.1023/B:PREV.0000013980.12412.cd 15058911

[7] Rathod S Gega L Degnan A Pikard J Khan T Husain N. The current status of culturally adapted mental health interventions: a practice-focused review of meta-analyses. Neuropsychiatr Dis Treat. (2018) 14:165–78. 10.2147/NDT.S138430 29379289 PMC5757988

[8] Waller G Turner H. Therapist drift redux: Why well-meaning clinicians fail to deliver evidence-based therapy, and how to get back on track. Behav Res Ther. (2016) 77:129–37. 10.1016/j.brat.2015.12.005 26752326

[9] Kirk MA Moore JE Wiltsey Stirman S Birken SA. Towards a comprehensive model for understanding adaptations' impact: The model for adaptation design and impact (MADI). Implement Sci. (2020) 15:1–15. 10.1186/s13012-020-01021-y 32690104 PMC7370455

[10] Moore JE Bumbarger BK Cooper BR. Examining adaptations of evidence-based programs in natural contexts. J Prim Prev. (2013) 34:147–61. 10.1007/s10935-013-0303-6 23605294

[11] Kakeeto M Lundmark R Hasson H von Thiele Schwarz U. Meeting patient needs trumps adherence. A cross-sectional study of adherence and adaptations when national guidelines are used in practice. J Eval Clin Pract. (2017) 23:830–8. 10.1111/jep.12726 28251758

[12] Aarons GA. Measuring Provider Attitudes Toward Evidence-Based Practice: Consideration of Organizational Context and Individual Differences. Child Adolesc Psychiatr Clin N Am. (2005) 14:255–viii. 10.1016/j.chc.2004.04.008 15694785 PMC1564127

[13] Finne J. Evidence-based practice in social work: who are the critics? J Soc Work. (2021) 21:1433–49. 10.1080/14780887.2020.1769238

[14] Rye M Torres EM Friborg O Skre I Aarons GA. The evidence-based practice attitude scale-36 (EBPAS-36): a brief and pragmatic measure of attitudes to evidence-based practice validated in US and Norwegian samples. Implement Sci. (2017) 12:1–11. 10.1186/s13012-017-0573-0 28372587 PMC5379724

[15] Hill LG Maucione K Hood BK. A focused approach to assessing program fidelity. Prev Sci. (2007) 8:25–34. 10.1007/s11121-006-0051-4 16967341

[16] Hasson H Leviton L Von Thiele Schwarz U. A typology of useful evidence: Approaches to increase the practical value of intervention research. BMC Med Res Methodol. (2020) 20:1–16. 10.1186/s12874-020-00992-2 32460833 PMC7254642

[17] Stirman SW Miller CJ Toder K Calloway A. Development of a framework and coding system for modifications and adaptations of evidence-based interventions. Implement Sci. (2013) 8:1–12. 10.1186/1748-5908-8-65 23758995 PMC3686699

[18] Mejia A Leijten P Lachman JM Parra-Cardona JR. Different Strokes for Different Folks? Contrasting approaches to cultural adaptation of parenting interventions. Prev Sci. (2017) 18:630–9. 10.1007/s11121-016-0671-2 27338569

[19] Kumpfer K Magalhães C Xie J. Cultural adaptation and implementation of family evidence-based interventions with diverse populations. Prev Sci. (2017) 18:649–59. 10.1007/s11121-016-0719-3 27757773

[20] Scourfield J Nasiruddin Q. Religious adaptation of a parenting programme: process evaluation of the family links Islamic values course for muslim fathers. Child Care Health Dev. (2015) 41:697–703. 10.1111/cch.12228 25649634 PMC4964912

[21] von Thiele Schwarz U Lyon AR Pettersson K Giannotta F Liedgren P Hasson H. Understanding the value of adhering to or adapting evidence-based interventions: a study protocol of a discrete choice experiment. Implementat Sci Commun. (2021) 2:88. 10.1186/s43058-021-00187-w 34380575 PMC8356451

[22] Ulfsdotter M Enebrink P Lindberg L. Effectiveness of a universal health-promoting parenting program: a randomized waitlistcontrolled trial of all children in focus. BMC Public Health. (2014) 14:1–11. 10.1186/1471-2458-14-1083 25326710 PMC4210619

[23] Kling A Forster M Sundell K Melin L. A randomized controlled effectiveness trial of parent management training with varying degrees of therapist support. Behav Ther. (2010) 41:530–42. 10.1016/j.beth.2010.02.004 21035616

[24] Sanders MR Kirby JN Tellegen CL Day JJ. The triple P-positive parenting program: a systematic review and meta-analysis of a multi-level system of parenting support. Clin Psychol Rev. (2014) 34:337–57. 10.1016/j.cpr.2014.04.003 24842549

[25] Cunningham C. Large group, community based, family-centered parent training. In: Barkley RA Murphy KR editors. Attention Deficit Hyperactivity Disorder: A Clinical Workbook. New York, NY: Guilford Press (2005). p. 480–98.

[26] Moretti M Holland R Moore K McKay S. An attachment-based parenting program for caregivers of severely conduct disordered adolescents: Preliminary findings. J Child Youth Care Work. (2004) 19:170–9.

[27] Council NR. Medicine Io. Preventing Mental, Emotional, and Behavioral Disorders Among Young People: Progress and Possibilities. Washington, DC: The National Academies Press (2009). 20662125

[28] Palinkas LA Horwitz SM Green CA Wisdom JP Duan N Hoagwood K. Purposeful sampling for qualitative data collection and analysis in mixed method implementation research. Adm Policy Ment Health. (2015) 42:533–44. 10.1007/s10488-013-0528-y 24193818 PMC4012002

[29] Von Thiele Schwarz U Förberg U Sundell K Hasson H. Colliding ideals - An interview study of how intervention researchers address adherence and adaptations in replication studies. BMC Med Res Methodol. (2018) 18:1–11. 10.1186/s12874-018-0496-8 29739337 PMC5941334

[30] Braun V Clarke V. Using thematic analysis in psychology. Qual Res Psychol. (2006) 3:77–101. 10.1191/1478088706qp063oa

[31] Braun V Clarke V. One size fits all? What counts as quality practice in (reflexive) thematic analysis? Qual Res Psychol. (2020) 18:328–52. 10.1177/1468017320955131

[32] Topolinski S Hertel G. The role of personality in psychotherapists' careers: Relationships between personality traits, therapeutic schools, and job satisfaction. Psychother Res. (2007) 17:365–75. 10.1080/10503300600830736

[33] Wiltsey Stirman S Gutner CA Crits-Christoph P Edmunds J Evans AC Beidas RS. Relationships between clinician-level attributes and fidelity-consistent and fidelity-inconsistent modifications to an evidence-based psychotherapy. Implement Sci. (2015) 10:115. 10.1186/s13012-015-0308-z 26268633 PMC4534152

[34] Barrera M Berkel C Castro FG. Directions for the advancement of culturally adapted preventive interventions: local adaptations, engagement, and sustainability. Prev Sci. (2017) 18:640–8. 10.1007/s11121-016-0705-9 27591993 PMC7678089

[35] Finno-Velasquez M Fettes DL Aarons GA Hurlburt MS. Cultural adaptation of an evidence-based home visitation programme: Latino clients' experiences of service delivery during implementation. J Child Serv. (2014) 9:280–94. 10.1108/JCS-06-2014-0030

[36] Gardner F Montgomery P Knerr W. Transporting evidence-based parenting programs for child problem behavior (Age 3–10) between countries: systematic review and meta-analysis. J Clin Child Adol Psychol. (2016) 45:749–62. 10.1080/15374416.2015.1015134 25785902

[37] Morawska A Sanders M Goadby E Headley C Hodge L McAuliffe C. Is the triple P-positive parenting program acceptable to parents from culturally diverse backgrounds? J Child Fam Stud. (2011) 20:614–22. 10.1007/s10826-010-9436-x22950888

[38] Haerpfer C Inglehart R Moreno A Welzel C Kizilova K Diez-Medrano J. World Values Survey: Round Seven – Country-Pooled Datafile. Madrid; Vienna:. JD Systems Institute & WVSA Secretariat (2020).

[39] Trifan TA Stattin H Tilton-Weaver L. Have authoritarian parenting practices and roles changed in the last 50 years? J Marriage Fam. (2014) 76:744–61. 10.1111/jomf.12124

[40] Lansford JE Alampay LP Al-Hassan S Bacchini D Bombi AS Bornstein MH. Corporal punishment of children in nine countries as a function of child gender and parent gender. Int J Pediatr. (2010) 2010:672780. 10.1155/2010/672780 20976255 PMC2952896

[41] Finkelhor D Turner H Wormuth BK Vanderminden J Hamby S. Corporal punishment: current rates from a National Survey. J Child Fam Stud. (2019) 28:1991–7. 10.1007/s10826-019-01426-434162281

[42] Janson S Jernbro C Långberg B. Kroppslig bestraffning och annan kränkning av barn i Sverige: en nationell kartläg- gning 2011. Karlstad: Karlstads Universitet (2011).

[43] Morrow SL. Quality and trustworthiness in qualitative research in counseling psychology. J Couns Psychol. (2005) 52:250–60. 10.1037/0022-0167.52.2.250

[44] Jutengren G Palmérus K. A comparison of Swedish and US fathers' self-reported use of parental discipline. Child Soc. (2002) 16:246–59. 10.1002/chi.708

[45] Escoffery C Lebow-Skelley E Udelson H Böing EA Wood R Fernandez ME. A scoping study of frameworks for adapting public health evidence-based interventions. Transl Behav Med. (2019) 9:1–10. 10.1093/tbm/ibx067 29346635 PMC6305563

[46] Chambers DA Glasgow RE Stange KC. The dynamic sustainability framework: Addressing the paradox of sustainment amid ongoing change. Implementation Science. (2013) 8:1–11. 10.1186/1748-5908-8-117 24088228 PMC3852739

[47] von Thiele Schwarz U Aarons GA Hasson H. The Value Equation: Three complementary propositions for reconciling fidelity and adaptation in evidence-based practice implementation. BMC Health Serv Res. (2019) 19:1–10. 10.1186/s12913-019-4668-y 31752846 PMC6873662

[48] Boswell JF. Monitoring processes and outcomes in routine clinical practice: a promising approach to plugging the holes of the practice-based evidence colander. Psychother Res. (2020) 30:829–42. 10.1080/10503307.2019.1686192 31672104

[49] Green LW. Making research relevant: if it is an evidence-based practice, where's the practice-based evidence? Fam Pract. (2008) 25(Suppl. 1):i20–4. 10.1093/fampra/cmn055 18794201

